# Supplementary material for: Panel strain of Klebsiella pneumoniae for beta-lactam antibiotic evaluation: their phenotypic and genotypic characterization
Source: PeerJ. 2017 Jan 19;5:e2896. doi: 10.7717/peerj.2896 (PMC5251932; doi:10.7717/peerj.2896)
Supplement: Supplemental Information 1 [file peerj-05-2896-s001.docx]

Table S1: Primers used for this study

|  | Target/purpose | Primer Sequence (Fwd-Rev; 5'-3') | Reference |
| --- | --- | --- | --- |
| IMP | Screening of MBL | CATGGTTTGGTGGTTCTTGT-ATAATTTGGCGGACTTTGGC | ([Patzer et al. 2009](#_ENREF_10)). |
| VIM | Screening of MBL | CACTTCTCGCGGAGATTGAA-GTGCTTTGACAACGTTCGCT | ([Patzer et al. 2009](#_ENREF_10)). |
| SIM | Screening of MBL | TACAAGGGATTCGGCATCG-TAATGGCCTGTTCCCATGTG | ([Patzer et al. 2009](#_ENREF_10)). |
| SPM | Screening of MBL | CTGCTTGGATTCATGGGCGC-CCTTTTCCGCGACCTTGATC | ([Toleman et al. 2002](#_ENREF_16)) |
| GIM | Screening of MBL | AACTTCCAACTTTGCCATGC-TCGACACACCTTGGTCTGAA | ([Patzer et al. 2009](#_ENREF_10)) |
| NDM | Screening of MBL | GGTTTGGCGATCTGGTTTTC-CGGAATGGCTCATCACGATC | ([Yong et al. 2009](#_ENREF_20)) |
| AIM | Screening of MBL | ATGAAACGTCGCTTCACCCTGCTG-TCAAGGCCGCGCGCCGCTGG | ([Leiros et al. 2012](#_ENREF_8)) |
| KHM | Screening of MBL | GGTATGCGCTGACGATTCAT-TACCGCATGGCTAAGATTGC | ([Sekiguchi et al. 2008](#_ENREF_14)) |
| KPC | Screening of Carbapenemases | TGTCACTGTATCGCCGTC-CTCAGTGCTCTACAGAAAACC | ([Yangsoon Lee 2014](#_ENREF_19)) |
| SME | Screening of Carbapenemases | AGCGGTTCCCTTTATGCAGT-CGTGATGCTTCCGCAATAGT | ([Queenan et al. 2000](#_ENREF_12)) |
| GES | Screening of Carbapenemases | CTTCATTCACGCACTATTAC-TAACTTGACCGACAGAGG | ([Poirel et al. 2000](#_ENREF_11)) |
| CMY-1 | Screening of AmpC | GGGGCATATGATGAAAAAATCGTTA-CCGGATCCTCAACCGGCCA | ([Bauernfeind et al. 1996](#_ENREF_3)) |
| CMY-2 | Screening of AmpC | AACACACTGATTGCGTCTGA-TCCTGGGCCTCATCGTCAGTTAT | ([Bauernfeind et al. 1996](#_ENREF_3)) |
| DHA-1 | Screening of AmpC | GCAAAGCCAGTATGCGTACG-CAGTTGTTGCGCCCGTTTTA | ([Verdet et al. 2006](#_ENREF_18)) |
| FOX | Screening of AmpC | TAGTCTGGGCCAGCCATTTG-GTAACCGGATTGGCCTGGAA | ([Gonzalez Leiza et al. 1994](#_ENREF_7)) |
| MIR | Screening of AmpC | TGACGACGCGGGTCTTTAAA-TAAATGCCACGTAGCTGCCA | ([Papanicolaou et al. 1990](#_ENREF_9)) |
| ACC-1 | Screening of AmpC | CCTCCGTCAGCTCAGATACA-TTTACTAGGTGCAAGCCAGACA | ([Bauernfeind et al. 1999](#_ENREF_2)) |
| LAT | Screening of AmpC | TGCCGTTATCTACCAGGGGA-TTCGTTCTGCGGAACCGTAA | ([Tzouvelekis et al. 1993](#_ENREF_17)) |
| ACT-1 | Screening of AmpC | CGTTACGCCGCTGATGAAAG-CCAGGGTAAGGCCTTTCCTG | ([Reisbig MD 2002](#_ENREF_13)) |
| ISAba | Screening of Insertion Sequence | CACGAATGCAGAAGTTG-CGACGAATACTATGACAC | ([Chen et al. 2010](#_ENREF_5)). |
| TEM | Screening of ESBL | GACAGTTACCAATGCTTAATC-ATAAAATTCTTGAAGACGAAA | ([C. Kamatchi 2009](#_ENREF_4)) |
| CTX-M14 | Screening of ESBL | CGCTTTGCGATGTGCAG-ACCGCGATATCGTTGGT | ([Dutour et al. 2002](#_ENREF_6)) |
| OXA | Screening of ESBL | CGCAAATGGCACCAGATTCA-TGTATGATTGCTGTTCCAGAT | ([Sugumar et al. 2014](#_ENREF_15)). |
| SHV-full | Screening of ESBL | ATTTGTCGCTTCTTTACTCGC-TTTATGGCGTTACCTTTGACC | ([Ahmed et al. 2013](#_ENREF_1)) |

Table S2: References used to confirm the resistance genes.

| **Resistant genes** | **Panel strains** | **Reference accession number (GenBank)** |
| --- | --- | --- |
| DHA-1 | YMC2011/11/B7578, YMC2010/8/B2027, YMC2012/8/C631 | Y16410 |
| CMY-2 | YMC2010/8/B2027 | DQ478727 |
| CTX-M-15 | YMC2011/7/B774, YMC2013/7/B3993, YMC2010/8/B2027 | DQ302097 |
| IMP-1 | YMC2012/8/C631 | AB469046 |
| OXA-1 | YMC2011/7/B774, YMC2011/7/B7207 | J02967 |
| OXA-9 | YMC2013/7/B3993 | JF703130 |
| SHV-11 | YMC2011/7/B774, YMC2010/8/B2027, YMC2011/8/B10311 | X98101 |
| SHV-12 | YMC2013/7/B3993, YMC2011/11/B7578 | AJ920369 |
| SHV-158 | YMC2011/11/B7578 | JX121125 |
| SHV-187 | YMC2011/7/B7207 | LN515533 |
| TEM-1 | YMC2011/7/B774, YMC2013/7/B3993, YMC2011/7/B7207, YMC2010/8/B2027, YMC2012/8/C631, YMC2011/8/B10311 | J01749 |
| aac(6’)-Ib | YMC2013/7/B3993 | M21682 |
| Aac(6’)-IIa | YMC2011/7/B774, YMC2011/7/B7207, YMC2010/8/B2027, YMC2012/8/C631 | M29695 |
| aadA1 | YMC2013/7/B3993 | JQ480156 |
| aadA2 | YMC2011/11/B7578 | JQ364967 |
| armA | YMC2011/11/B7578 | AY220558 |
| strA | YMC2011/7/B774, YMC2013/7/B3993, YMC2011/7/B7207, YMC2011/11/B7578, YMC2010/8/B2027, YMC2012/8/C631 | NC_003384 |
| strB | YMC2011/7/B774, YMC2013/7/B3993, YMC2011/7/B7207, YMC2011/11/B7578, YMC2010/8/B2027, YMC2012/8/C631 | M96392 |
| QnrB66 | YMC2011/7/B774, YMC2013/7/B3993, YMC2011/7/B7207 | KC580655 |
| QnrB4 | YMC2011/11/B7578, YMC2010/8/B2027 | DQ303921 |
| aac(6’)Ib-cr | YMC2011/7/B774, YMC2013/7/B3993, YMC2011/7/B7207, YMC2010/8/B2027 | DQ303918 |
| oqxA | YMC2013/7/B3993, YMC2011/7/B7207, YMC2011/11/B7578, YMC2011/8/B10311 | EU370913 |
| oqxB | YMC2011/7/B774, YMC2013/7/B3993, YMC2011/7/B7207, YMC2011/11/B7578, YMC2011/8/B10311 | EU370913 |
| catA2 | YMC2010/8/B2027 | X53796 |
| catB3 | YMC2011/7/B774, YMC2011/7/B7207 | AJ009818 |
| tet(A) | YMC2011/7/B774, YMC2011/7/B7207, YMC2011/8/B10311 | AJ517790 |
| dfrA14 | YMC2011/7/B774, YMC2013/7/B3993, YMC2011/7/B7207, YMC2010/8/B2027, YMC2011/8/B10311 | DQ388123 |
| Sul1 | YMC2011/11/B7578, YMC2010/8/B2027, YMC2012/8/C631 | CP002151 |
| Sul2 | YMC2011/7/B774, YMC2013/7/B3993, YMC2011/7/B7207, YMC2012/8/C631 | GQ421466 |

Table S3: Complete list of panel strains and its MIC.

| **Strains** | **PIP** | **PIP+TZ** | **CAZ** | **FEP** | **AZT** | **IMI** | **MER** | **CIP** | **CAZ/CLV** | **FOX** | **AMP** | **SAM** |
| --- | --- | --- | --- | --- | --- | --- | --- | --- | --- | --- | --- | --- |
| **ESBL** | R |  | R | R |  | S | S |  | S | S | R | V |
| YMC2011/7/B774 | 256(R) | 32(I) | 256(R) | 128(R) | 128(R) | 0.25(S) | 0.25(S) | 16(R) | 1(S) | *32(R)* | 256(R) | 64(R) |
| YMC2013/7/B3993 | 256(R) | 128(R) | 256(R) | 64(R) | 128(R) | 0.25(S) | 0.25(S) | 128(R) | 1(S) | *32(R)* | 256(R) | 128(R) |
| YMC2011/7/B7207 | 256(R) | 32(I) | 64(R) | *16(I)* | 128(R) | 0.25(S) | 0.25(S) | 256(R) | 1(S) | *32(R)* | 256(R) | 64(R) |
| YMC2011/11/B7578 | 256(R) | 32(I) | 256(R) | 64(R) | 128(R) | 64(R) | 1(S) | 128(R) | 1(S) | *128(R)* | 256(R) | 64(R) |
| YMC2011/11/B10514 | 256(R) | 32(I) | 256(R) | 64(R) | 128(R) | *2(I)* | 0.25(S) | 32(R) | *128(R)* | *256(R)* | 256(R) | 128(R) |
| YMC2010/08/B4822 | 256(R) | 128(I) | 256(R) | *4(S)* | 128(R) | 0.25(S) | 0.25(S) | 128(R) | *32(R)* | *256(R)* | 256(R) | 128(R) |
| **High level ampc β-lactamase** | R | R | R | S |  | S | S |  | R | *R* | R | R |
| YMC2010/8/B2027 | 256(R) | 128(R) | 256(R) | 2(S) | 128(R) | 0.5(S) | 0.25(S) | 2(I) | 64(R) | 256(R) | 256(R) | 128(R) |
| **Carbapenemase** | R | R | R | R |  | R | R |  | R | R | R | R |
| YMC2012/8/C631 | 256(R) | *8(S)* | 256(R) | 32(R) | 16(I) | 64(R) | 64(R) | 1(S) | 32(R) | 256(R) | 256(R) | 128(R) |
| YMC2010/08/C782 | 256(R) | *8(S)* | 256(R) | *16(I)* | 16(I) | 64(R) | 64(R) | 1(S) | 32(R) | 256(R) | 256(R) | 64(R) |
| YMC2012/09/S50 | 256(R) | *8(S)* | 256(R) | 32(R) | 16(I) | 64(R) | 16(R) | 1(S) | 32(R) | 256(R) | 256(R) | 128(R) |
| YMC2012/09/S50 | 256(R) | 128(R) | 256(R) | *8(S)* | 128(R) | 4(R) | 4(R) | 64(R) | 128(R) | 256(R) | 256(R) | 128(R) |
| YMC2013/03/R1024 | 256(R) | 128(R) | 64(R) | *2(S)* | 0.25(S) | *1(S)* | *2(I)* | 0.5(S) | 32(R) | 256(R) | 256(R) | 128(R) |
| YMC2011/10/B2570 | 256(R) | 128(R) | 256(R) | 32(R) | 16(I) | 4(R) | 4(R) | 4(R) | 64(R) | 256(R) | 256(R) | 128(R) |
| YMC2011/10/B1822 | 256(R) | *2(S)* | 256(R) | 32(R) | 16(I) | 32(R) | 16(R) | 2(I) | 128(R) | 256(R) | 256(R) | 128(R) |
| YMC2013/1/KU2 | 256(R) | *8(S)* | 256(R) | 128(R) | 32(R) | 256(R) | 128(R) | 64(R) | 64(R) | 256(R) | 256(R) | 128(R) |
| YMC2013/1/NDM-506 | 256(R) | 128(R) | 256(R) | 128(R) | 128(R) | 256(R) | 128(R) | 128(R) | 128(R) | 256(R) | 256(R) | 128(R) |
| YMC2014/3/MP14 | 256(R) | 128(R) | 64(R) | 32(R) | 128(R) | 32(R) | 32(R) | 64(R) | 128(R) | 64(R) | 256(R) | 128(R) |
| **High level acquired penicillinase** | R |  | S | S |  | S | S |  | S | S | R | R |
| YMC2011/8/B10311 | 256(R) | 4(S) | 2(S) | 4(S) | 2(S) | 0.5(S) | *2(I)* | 0.5(S) | 2(S) | 8(S) | 256(R) | 128(R) |
| **Wild type** | I | S | S | S |  | S | S |  | S | S | R | S |
| YMC2013/12/R3191 | 4(S) | 1(S) | 0.5(S) | 0.03(S) | 0.25(S) | 0.25(S) | 0.25(S) | 0.12(S) | 0.5(S) | 2(S) | 64(R) | *16(I)* |

Note: Note: MLST, Multilocus sequence typing; R, Resistant; I, Intermediate; S, susceptible; V, Variable; PIP, Piperacillin; PIP/TZ, Piperacillin/Tazobactam; CAZ,ceftazidime; FEP, cefepime; AZT, Aztreonam; IMI, Imipenem; MER, Meropenem; CIP, Ciprofloxacin, CAZ/CLV, ceftazidime/clavulanate; FOX, cefoxitin; AMP, Ampicillin; SAM, Ampicillin/Sulbactam.

Table S4: Raw sequence data from Ion Torrent PGM Sequencer of the panel strains.

| **Strains** | **Bases** | **≥Q20bases** | **Reads** | **Mean read length** |
| --- | --- | --- | --- | --- |
| **ESBL** |  |  |  |  |
| YMC2011/7/B774 | 155,676,588 | 130,371,915 | 720,463 | 216 |
| YMC2013/7/B3993 | 205,100,384 | 172,553,864 | 911,427 | 225 |
| YMC2011/7/B7207 | 180,282,790 | 154,674,935 | 890,939 | 202 |
| YMC2011/11/B7578 | 182,887,905 | 151,370,906 | 721,072 | 254 |
| **High level Ampc**  **β-lactamase** |  |  |  |  |
| YMC2010/8/B2027 | 248,533,006 | 207,231,256 | 1,125,343 | 221 |
| **Carbapenemase** |  |  |  |  |
| YMC2012/8/C631 | 210,002,265 | 174,711,461 | \|  \| 977,292 \| \| --- \| --- \| | 215 |
| **High level acquired penicillinase** |  |  |  |  |
| YMC2011/8/B10311 | 157,618,861 | 137,135,462 | \|  \| 780,323 \| \| --- \| --- \| | 202 |

*all the units are in base-pairs.

Table S5: Subsystem information for the panel strains.

| Strains | Cofactors, Vitamins, Prosthetic Groups, Pigments | Cell Wall and Capsule | Virulence, Disease and Defense | Potassium metabolism | Phages, Prophages, Transposable elements, Plasmids | Membrane Transport | Iron acquisition and metabolism | RNA Metabolism | Nucleosides and Nucleotides | Protein Metabolism | Cell Division and Cell Cycle | Motility and Chemotaxis | Regulation and Cell signaling | Secondary Metabolism | DNA Metabolism | Fatty Acids, Lipids, and Isoprenoids | Nitrogen Metabolism | Dormancy and Sporulation | Respiration | Stress Response | Metabolism of Aromatic Compounds | Amino Acids and Derivatives | Sulfur Metabolism | Phosphorus Metabolism | Carbohydrates |
| --- | --- | --- | --- | --- | --- | --- | --- | --- | --- | --- | --- | --- | --- | --- | --- | --- | --- | --- | --- | --- | --- | --- | --- | --- | --- |
| **ESBL** |  |  |  |  |  |  |  |  |  |  |  |  |  |  |  |  |  |  |  |  |  |  |  |  |  |
| YMC2011/7/B774 | 323 | 206 | 148 | 36 | 32 | 262 | 67 | 228 | 145 | 268 | 41 | 9 | 155 | 5 | 168 | 134 | 47 | 5 | 183 | 182 | 78 | 507 | 77 | 64 | 858 |
| YMC2013/7/B3993 | 328 | 218 | 146 | 34 | 69 | 365 | 68 | 224 | 143 | 296 | 45 | 9 | 165 | 5 | 173 | 132 | 47 | 5 | 178 | 178 | 79 | 518 | 76 | 62 | 883 |
| YMC2011/7/B7207 | 326 | 209 | 128 | 34 | 64 | 209 | 67 | 222 | 143 | 301 | 37 | 11 | 151 | 5 | 146 | 126 | 47 | 5 | 176 | 176 | 79 | 520 | 75 | 62 | 840 |
| YMC2011/11/B7578 | 327 | 207 | 129 | 35 | 65 | 253 | 69 | 224 | 141 | 280 | 43 | 8 | 162 | 5 | 110 | 140 | 47 | 5 | 181 | 180 | 78 | 517 | 76 | 62 | 875 |
| **High level Ampc β-lactamase** |  |  |  |  |  |  |  |  |  |  |  |  |  |  |  |  |  |  |  |  |  |  |  |  |  |
| YMC2010/8/B2027 | 337 | 229 | 153 | 46 | 42 | 289 | 68 | 230 | 144 | 314 | 47 | 10 | 171 | 18 | 204 | 131 | 50 | 6 | 195 | 197 | 78 | 520 | 78 | 62 | 850 |
| **Carbapenemase** |  |  |  |  |  |  |  |  |  |  |  |  |  |  |  |  |  |  |  |  |  |  |  |  |  |
| YMC2012/8/C631 | 325 | 200 | 139 | 37 | 47 | 366 | 69 | 229 | 141 | 296 | 44 | 9 | 155 | 5 | 176 | 128 | 47 | 6 | 180 | 175 | 79 | 500 | 77 | 60 | 819 |
| **High level acquired penicillinase** |  |  |  |  |  |  |  |  |  |  |  |  |  |  |  |  |  |  |  |  |  |  |  |  |  |
| YMC2011/8/B10311 | 329 | 207 | 150 | 34 | 46 | 227 | 77 | 226 | 144 | 300 | 41 | 11 | 165 | 5 | 144 | 128 | 46 | 5 | 177 | 178 | 79 | 520 | 76 | 62 | 868 |

Table S6: Mutations in OmpK36 gene in YMC2011/7/B7207, YMC2011/8/B10311 and YMC2011/7/B774.

| Ser88-Gly |
| --- |
| Ser90-Asp |
| Asp91-Lys |
| Gly181-Del |
| Ala183-Del |
| Leu184-Del |
| Ser185-Asp |
| Pro186-Met |
| Thr192-Gly |
| Leu194-Glu |
| Tyr201-Phe |
| Leu205-Val |
| Tyr210-Trp |
| Leu225-Thr |
| Gly226-Asp |
| Lys231-Del |
| Ala233-Val |
| Asn239-Asp |
| Thr258-Ser |
| Arg309-Add |
| 350His-Arg |

Figure S1: The SDS-Page image of the panel strains representing the porins.


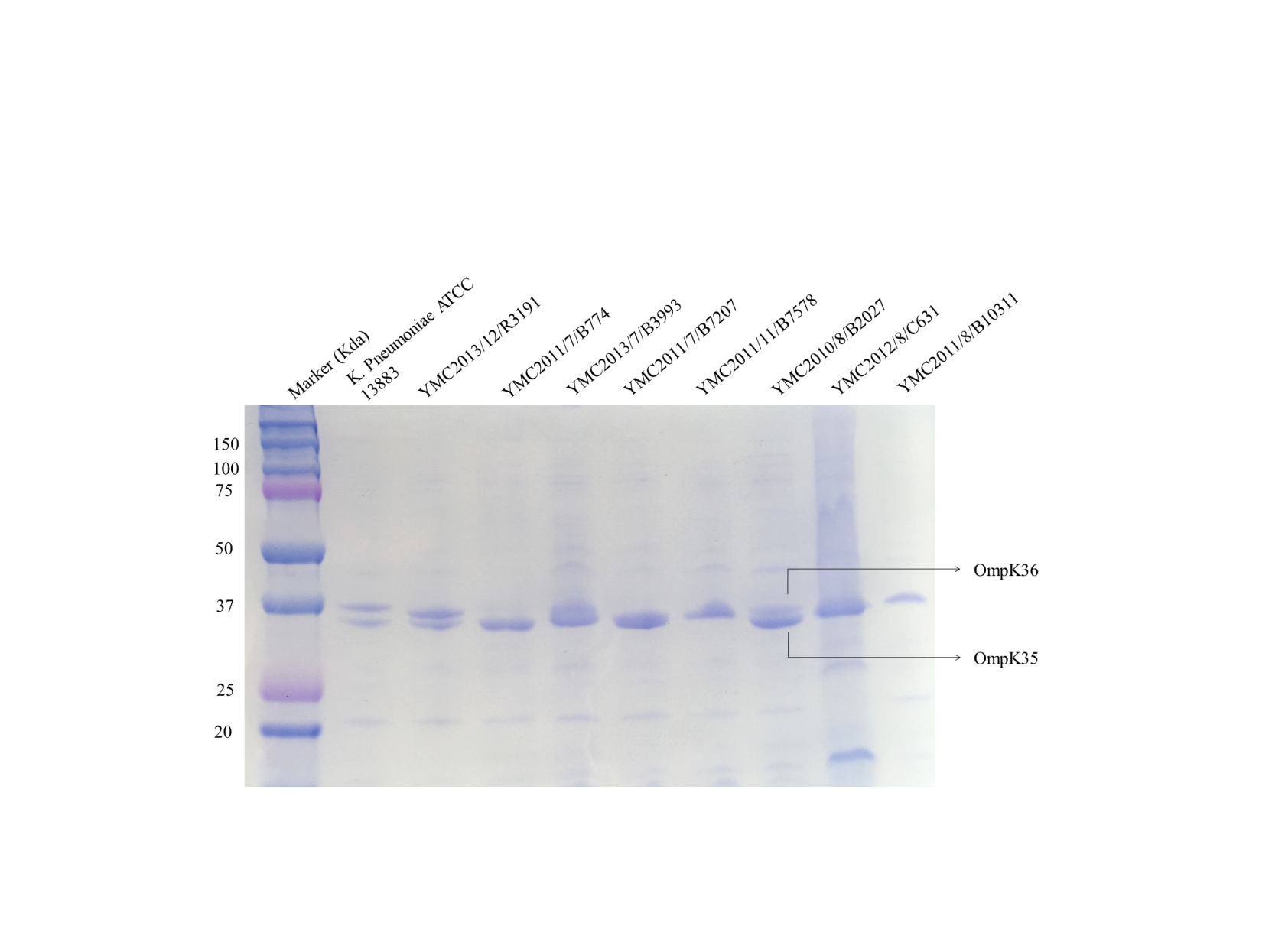


Figure S2: Nucleotide alignment of OmpK 35 gene of panel strain: OmpK-35 gene of YMC2013/7/B3993 and OmpK-35_YMC2012/8/C631 has been interrupted by insertion of transposon elements (Highlighted in yellow). Arrows indicate the start codon, RBS, -10, -35 and transcription regulators.

1 -------------------------------------GTGTAACGCTTACTGATG---AC OmpK-35_YMC2013/7/B3993

1 AACAGATTACAACCGTCGCTCGATAGCGGAAACGGCGATGTACCGGGTAAAACAGCTGTT OmpK-35_YMC2012/8/C631

1 -----GTT------------------------ACGCACTGTTTCGGTGCATCCTGGTGAT OmpK-35_YMC2011/7/B774

1 -----GTT------------------------ACGCACTGTTTCGGTGCATCCTGGTGAT OmpK-35_YMC2010/8/B2027

1 -----GTT------------------------ACGCACTGTTTCGGTGCATCCTGGTGAT OmpK-35_YMC2011/11/B7578

1 -----GTT------------------------ACGCACTGTTTCGGTGCATCCTGGTGAT OmpK-35_YMC2011/7/B7207

1 -----GTT------------------------ACGCACTGTTTCGGTGCATCCTGGTGAT OmpK-35_YMC2011/8/B10311

*** ** *

21 GTGCAG----TTTTCCCTTCAGAC---------GTGACTCATACATCGGCCAGCCGTCCG OmpK-35_YMC2013/7/B3993

61 CGGGGGTTCACTGACGCTGC--------GTGACTACGATGGTCAGGTTGCGGAGGCTATG OmpK-35_YMC2012/8/C631

32 GTTGATCGCACTTTATGTTCATAACAGAGCAAAATGGCTGATAAATTTGCTCATTTTTTG OmpK-35_YMC2011/7/B774

32 GTAGATCGCACTTTATGTTCATAACTGAGCAAAACCGCTGATAAATTTGCTCATTTTTTG OmpK-35_YMC2010/8/B2027

32 GTAGATCGCACTTTATGTTCATAACTGAGCAAAACCGCTGATAAATTTGCTCATTTTTTG OmpK-35_YMC2011/11/B7578

32 GTAGATCGCACTTTATGTTCATAACTCAGCAAAACCGCTGATAAATTTGCTCATTTTTTG OmpK-35_YMC2011/7/B7207

32 GCAGATCGCACTTTATGTTCATAACTGAGCAAAACCGCTGATAAATTTGCTCATTTTTTG OmpK-35_YMC2011/8/B10311

* * * * * ** * *

68 TCATCCATACCACCACGTCAAAAACTGACAGCAGGCTCAGAAGACGCTCCAGTGTGGCCA OmpK-35_YMC2013/7/B3993

113 GCCCTGGTACGAGCGCTGAACAAAATGACGAAAGC---AGGTATGCCT-GAAAGCGTGCG OmpK-35_YMC2012/8/C631

92 ACCTTTACCCGCACATCTTGCAA--AGACA--------GAGACTGAATAAAAAGAATACG OmpK-35_YMC2011/7/B774

92 ACCTTTACCCGCACATCTTGCAA--AGACA--------GAGACTGAATAAAAAGAATACG OmpK-35_YMC2010/8/B2027

92 ACCTTTACCCGCACATCTTGCAA--AGACA--------GAGACTGAATAAAAAGAATACG OmpK-35_YMC2011/11/B7578

92 ACCTTTACCCGCACATCTTGCAA--AGACA--------GAGACTGAATAAAAAGAATACG OmpK-35_YMC2011/7/B7207

92 ACCTTTACCCGCACATCTTGCAA--AGACA--------GAGACTGAATAAAAAGAATACG OmpK-35_YMC2011/8/B10311

* * * ** *** * * * *

128 TAGTGCGTTCACCGAAAACGTGGGCTACCACCG-CCCTGCGTATCCTGTCATACGCGTAA OmpK-35_YMC2013/7/B3993

169 TATTACCTGAAAACACAACCCGCTACGGG------GGAGACTTACCTGAAATCTGATTTA OmpK-35_YMC2012/8/C631

142 AATTCAATACACAAAATGAACGAATTGCCGCTTAGCCAAATTAATCATAAATAATTCATA OmpK-35_YMC2011/7/B774

142 AATTCAATACACAAAATGAACGAATTGCCGCTTAGCCAAATTAATCATAAATAATTCATA OmpK-35_YMC2010/8/B2027

142 AATTCAATACACAAAATGAACGAATTGCCGCTTAGCCAAATTAATCATAAATAATTCATA OmpK-35_YMC2011/11/B7578

142 AATTCAATACACAAAATGAACGAATTGCCGCTTAGCCAAATTAATCATAAATAATTCATA OmpK-35_YMC2011/7/B7207

142 AATTCAATACACAAAATGAACGAATTGCCGCTTAGCCAAATTAATCATAAATAATTCATA OmpK-35_YMC2011/8/B10311

* * * * * * * * ** *

187 AACAGCCAGCGCTGACGTGATTTT----GCACCGACGTAGCCCCATTGCTCGTCCATCTC OmpK-35_YMC2013/7/B3993

223 TTCAACAAAGCCTGAACGAGTTCGT----------------------------------- OmpK-35_YMC2012/8/C631

202 TAGATAGATAAGTAATGGCGTTTGCCCTTATCCGGTGGAACGGAATTT------------ OmpK-35_YMC2011/7/B774

202 TAGATAGATAAGTAATGGCGTTTGCCCTTATCCGGTGGAACGGAATTT------------ OmpK-35_YMC2010/8/B2027

202 TAGATAGATAAGTAATGGCGTTTGCCCTTATCCGGTGGAACGGAATTT------------ OmpK-35_YMC2011/11/B7578

202 TAGATAGATAAGTAATGGCGTTTTCCCTTATCCGGTGGAACGGAATTT------------ OmpK-35_YMC2011/7/B7207

202 TAGATAGATAAGTAATGGCGTTTGCCCTTATCCGGTGGAACGGAATTT------------ OmpK-35_YMC2011/8/B10311

IHF * * * **

243 CGCACAGACAATGACATCACTGCCCGGCTGTATCCGGGAGGTTACTGACTGCGGCCTGAG OmpK-35_YMC2013/7/B3993

248 -------GCAATTCATTCAGGGCTTCTGAATCTCCCCACAGTATAAAACCTGCATTGTTC OmpK-35_YMC2012/8/C631

250 -------TGATTAAGTTCACAAAGTTCCGTTAAATACACATTTAGTTACATGATTTTTCG OmpK-35_YMC2011/7/B774

250 -------TGATTAAGTTCACAAAGTTCCGTTAAATACACATTTAGTTACATGATTTTTCG OmpK-35_YMC2010/8/B2027

250 -------TGATTAAGTTCACAAAGTTCCGTTAAATACACATTTAGTTACATGATTTTTCG OmpK-35_YMC2011/11/B7578

250 -------TGATTAAGTTCACAAAGTTCCGTTAAATACACATTTAGTTACATGATTTTTCG OmpK-35_YMC2011/7/B7207

250 -------TGATTAAGTTCACAAAGTTCCGTTAAATACACATTTAATTACATGATTTTTCG OmpK-35_YMC2011/8/B10311

* * *** * F1 * ** F3

303 TTTTTTAAATGGCGCAAAATGGTGTTGAGGCCCACGCCCATTAGTCGTGCGGTGGCACGG OmpK-35_YMC2013/7/B3993

301 GGCGTTAACTCGTATCGAAGCATGAGATCAATCATCCTGTTGTTTCAGTCGAAGGAAAGT OmpK-35_YMC2012/8/C631

303 TTTTGTTACTCATATGAGATATTCGTAG-----------CATTTTCCGGCTAGCGAAACG OmpK-35_YMC2011/7/B774

303 TTTTGTTACTCATATGAGATATTCGTAG-----------CATTTTCCGGCTAGCGAAACG OmpK-35_YMC2010/8/B2027

303 TTTTGTTACTCATATGAGATATTCGTAG-----------CATTTTCCGGCTAGCGAAACG OmpK-35_YMC2011/11/B7578

303 TTTTGTTACTCATATGAGATATTCGTAG-----------CATTTTCCGGCTAGCGAAACG OmpK-35_YMC2011/7/B7207

303 TTTTGTTACTCATATGAGATATTCGTAG-----------CATTTTCCGGCTAGCGAAACG OmpK-35_YMC2011/8/B10311

* * * * * F4 ** *-35 * *

363 CAACCGA----------CGCCGTTCATAGCCATATCAATGATTTTCTGGTGTGTACCGGG OmpK-35_YMC2013/7/B3993

361 CTATCAGCAAAGAGAATCTGAGATCAAAGTGGA---------CATTTTAATTGAGCCGGA OmpK-35_YMC2012/8/C631

352 TTGTCGCGGATGGAAAGATGCCTTCAGACACCAAACTCTCATCAATGGTTCTGTAAGTTT OmpK-35_YMC2011/7/B774

352 TTGTCGCGGATGGAAAGATGCCTTCAGACACCAAACTCTCATCAATGGTTCTGTAAGTTT OmpK-35_YMC2010/8/B2027

352 TTGTCGCGGATGGAAAGATGCCTTCAGACACCAAACTCTCATCAATGGTTCTGTAAGTTT OmpK-35_YMC2011/11/B7578

352 TTGTCGCGGATGGAAAGATGCCTTCAGACACCAAACTCTCATCAATGGTTCTGTAAGTTT OmpK-35_YMC2011/7/B7207

352 TTGTCGCGGATGGAAAGATGCCTTCAGACACCAAACTCTCATCAATGGTTCTGTAAGTTT OmpK-35_YMC2011/8/B10311

* -10 *** * **

413 CTGAGAAGCGGCATAAGTGAAGGTGAGCTGC------CATGTTTTACGGCAGTGAGAGCA OmpK-35_YMC2013/7/B3993

412 TAAAGGACATTTCAATTGAGCTTTGACAGCACGTGTTCATATAAAAAATATTAATGAGGG OmpK-35_YMC2012/8/C631

412 TTATTGACAGAACTTATTGACGGCAGTGGCACGTGTTCATATAAAAAATATTAATGAGGG OmpK-35_YMC2011/7/B774

412 TTATTGACAGAACTTATTGACGGCAGTGGCACGTGTTCATATAAAAAATATTAATGAGGG OmpK-35_YMC2010/8/B2027

412 TTATTGACAGAACTTTTTGACGGCAGTGGCACGTGTTCATATAAAAAATATTAATGAGGG OmpK-35_YMC2011/11/B7578

412 TTATTGACAGAACTTATTGACGGCAGTGGCACGTGTTCATATAAAAAATATTAATGAGGG OmpK-35_YMC2011/7/B7207

412 TTATTGACAGAACTTATTGACGGCAGTGGCACGTGTTCATATAAAAAATATTAATGAGGG OmpK-35_YMC2011/8/B10311

* *** * * ***

RBS

467 GAGATAACGTTGATGCCCGGCAGTACTTTTGCCATTACGCATCACGCCTTCAGTAGCGGA OmpK-35_YMC2013/7/B3993

472 TAATAAATAATGATGAAGCGCAATATTCTGGCAGTGGTGATCCCTGCCCTGCTGGTAGCC OmpK-35_YMC2012/8/C631

472 TAATAAATAATGATGAAGCGCAATATTCTTGCAGTGGTGATCCCTGCCCTGCTGGTAGCC OmpK-35_YMC2011/7/B774

472 TAATAAATAATGATGAAGCGCAATATTCTGGCAGTGGTGATCCCTGCCCTGCTGGTAGCC OmpK-35_YMC2010/8/B2027

472 TAATAAATAATGATGAAGCGCAATATTCTGGCAGTGGTGATCCCTGCCCTGCTGGTAGCC OmpK-35_YMC2011/11/B7578

472 TAATAAATAATGATGAAGCGCAATATTCTGGCAGTGGTGATCCCTGCCCTGCTGGTAGCC OmpK-35_YMC2011/7/B7207

472 TAATAAATAATGATGAAGCGCAATATTCTGGCAGTGGTGATCCCTGCCGTGCTGGTAGCC OmpK-35_YMC2011/8/B10311

* ** ***** *** ** * * ** * * * *** * *

Start

527 ACAGGAGGGACAGCAGACGGAGACTGAAGCCACGGGAGCACCTCAAAAACACCATTATAC OmpK-35_YMC2013/7/B3993

532 GGT------GCAGCCAACGCTGCAGAAATCTATAACAAAAACGGCA-ACAAACTGGACTT OmpK-35_YMC2012/8/C631

532 GGT------GCAGCCAACGCTGCAGAAATCTATAACAAAAACGGCA-ACAAACTGGACTT OmpK-35_YMC2011/7/B774

532 GGT------GCAGCCAACGCTGCAGAAATCTATAACAAAAACGGCA-ACAAACTGGACTT OmpK-35_YMC2010/8/B2027

532 GG-------GCAGCCAACGCTGCAGAAATCTATAACAAAAACGGCA-ACAAACTGGACTT OmpK-35_YMC2011/11/B7578

532 GGT------GCAGCCAACGCTGCAGAAATCTATAACAAAAACGGCA-ACAAACTGGACTT OmpK-35_YMC2011/7/B7207

532 GGT------GCAGCCAACGCTGCAGAAATCTATAACAAAAACGGCA-ACAAACTGGACTT OmpK-35_YMC2011/8/B10311

**** *** * ** * * * * * * * * * *

587 ACTAAATCAGCAAGTTGGTAGCATCACCTGGACCACCAATGGCGACACCAGCAGCGACGA OmpK-35_YMC2013/7/B3993

585 CTATGGAAAAATGGTCGGCGAGCACGTCTGGACCACCAATGGCGACACCAGCAGCGACGA OmpK-35_YMC2012/8/C631

585 CTATGGAAAAATGGTCGGCGAGCACGTCTGGACCACCAATGGCGACACCAGTAGCGACGA OmpK-35_YMC2011/7/B774

585 CTATGGAAAAATGGTCGGCGAGCACGTCTGGACCACCAATGGCGACACCAGCAGCGACGA OmpK-35_YMC2010/8/B2027

584 CTATGGAAAAATGGTCGGCGAGCACGTCTGGACCACCAATGGCGACACCAGCAGCGACGA OmpK-35_YMC2011/11/B7578

585 CTATGGAAAAATGGTCGGCGAGCACGTCTGGACCACCAATGGCGACACCAGCAGCGACGA OmpK-35_YMC2011/7/B7207

585 CTATGGAAAAATGGTCGGCGAGCACGTCTGGACCACCAATGGCGACACCAGCAGCGACGA OmpK-35_YMC2011/8/B10311

* ** ** * ************************ ********

647 TACCACCTATGCCCGTATCGGCCTGAAAGGCGAAACTCAGATCAACGATCAGCTGATCGG OmpK-35_YMC2013/7/B3993

645 TACCACCTATGCCCGTATCGGCCTGAAAGGCGAAACTCAGATCAACGATCAGCTGATCGG OmpK-35_YMC2012/8/C631

645 TACCACCTATGCCCGTATCGGCCTGAAAGGCGAAACTCAGATCAACGATCAGCTGATCGG OmpK-35_YMC2011/7/B774

645 TACCACCTATGCCCGTATCGGCCG-----GCGAAACTCAGATCAACGATCAGCTGATCGG OmpK-35_YMC2010/8/B2027

644 TACCACCTATGCCCGTATCGGCCTGAAAGGCGAAACTCAGATCAACGATCAGCTGATCGG OmpK-35_YMC2011/11/B7578

645 TACCACCTATGCCCGTATCGGCCTGAAAGGCGAAACTCAGATCAACGATCAGCTGATCGG OmpK-35_YMC2011/7/B7207

645 TACCACCTATGCCCGTATCGGCCTGAAAGGCGAAACTCAGATCAACGATCAGCTGATCGG OmpK-35_YMC2011/8/B10311

*********************** *******************************

707 CTACGGCCAGTGGGAATACAACATGGACGCGTCCAATGTTGAAGGTTCCCAGACCACAAA OmpK-35_YMC2013/7/B3993

705 CTACGGCCAGTGGGAATACAACATGGACGCGTCCAATGTTGAAGGTTCCCAGACCACAAA OmpK-35_YMC2012/8/C631

705 CTACGGCCAGTGGGAATACAACATGGACGCGTCCAATGTTGAAGGTTCCCAGACCACAAA OmpK-35_YMC2011/7/B774

700 CTACGGCCAGTGGGAATACAACATGGACGCGTCCAATGTTGAAGGTTCCCAGACCACAAA OmpK-35_YMC2010/8/B2027

704 CTACGGCCAGTGGGAATACAACATGGACGCGTCCAATGTTGAAGGTTCCCAGACCACAAA OmpK-35_YMC2011/11/B7578

705 CTACGGCCAGTGGGAATACAACATGGACGCGTCCAATGTTGAAGGTTCCCAGACCACAAA OmpK-35_YMC2011/7/B7207

705 CTACGGCCAGTGGGAATACAACATGGACGCGTCCAATGTTGAAGGTTCCCAGACCACAAA OmpK-35_YMC2011/8/B10311

************************************************************

767 AACCCGTCTAGCGTTCGCAGGCCTGAAAGCGGGCGAATACGGTTCATTCGACTATGGCCG OmpK-35_YMC2013/7/B3993

765 AACCCGTCTGGCGTTCGCGGGCCTGAAAGCGGGCGAATACGGTTCATTCGACTATGGCCG OmpK-35_YMC2012/8/C631

765 AACCCGTCTGGCGTTCGCGGGCCTGAAAGCGGGCGAATACGGTTCATTCGACTATGGCCG OmpK-35_YMC2011/7/B774

760 AACCCGTCTGGCGTTCGCGGGCCTGAAAGCGGGCGAATACGGTTCATTCGACTATGGCCG OmpK-35_YMC2010/8/B2027

764 AACCCGTCTAGCGTTCGCAGGCCTGAAAGCGGGCGAATACGGTTCATTCGACTATGGCCG OmpK-35_YMC2011/11/B7578

765 AACCCGTCTGGCGTTCGCAGGCCTGAAAGCGGGCGAATACGGTTCATTCGACTATGGCCG OmpK-35_YMC2011/7/B7207

765 AACCCGTCTGGCGTTCGCAGGCCTGAAAGCGGGCGAATACGGTTCATTCGACTATGGCCG OmpK-35_YMC2011/8/B10311

********* ******** *****************************************

827 TAACTACGGCGCGATCTACGACGTCGAAGCGGCAACCGATATGCTGGTTGAATGGGGCGG OmpK-35_YMC2013/7/B3993

825 TAACTACGGCGCGATCTACGACGTCGAAGCGGCAACCGATATGCTGGTTGAATGGGGCGG OmpK-35_YMC2012/8/C631

825 TAACTACGGCGCGATCTACGACGTCGAAGCGGCAACCGATATGCTGGTTGAATGGGGCGG OmpK-35_YMC2011/7/B774

820 TAACTACGGCGCGATCTACGACGTCGAAGCGGCAACCGATATGCTGGTTGAATGGGGCGG OmpK-35_YMC2010/8/B2027

824 TAACTACGGCGCGATCTACGACGTCGAAGCGGCAACCGATATGCTGGTTGAATGGGGCGG OmpK-35_YMC2011/11/B7578

824 TAACTATGGCGCGATCTACGACGTCGAAGCGGCAACCGATATGCTGGTTGAATGGGGCGG OmpK-35_YMC2011/7/B7207

825 TAACTACGGCGCGATCTACGACGTCGAAGCGGCAACCGATATGCTGGTTGAATGGGGCGG OmpK-35_YMC2011/8/B10311

****** *****************************************************

887 TGACGGCTGGAACTATACCGACAACTACATGACCGGTCGTACCAACGGCGTCGCAACCTA OmpK-35_YMC2013/7/B3993

885 TGACGGCTGGAACTACACCGACAACTACATGACCGGTCGTACCAACGGCGTCGCAACCTA OmpK-35_YMC2012/8/C631

885 TGACGGCTGGAACTACACCGACAACTACATGACCGGTCGTACCAACGGCGTCGCAACCTA OmpK-35_YMC2011/7/B774

880 TGACGGCTGGAACTACACCGACAACTACATGACCGGTCGTACCAACGGCGTCGCAACCTA OmpK-35_YMC2010/8/B2027

884 TGACGGCTGGAACTATACCGACAACTACATGACCGGTCGTACCAACGGCGTCGCAACCTA OmpK-35_YMC2011/11/B7578

885 TGACGGCTGGAACTACACCGACAACTACATGACCGGTCGTACCAACGGCGTCGCAACCTA OmpK-35_YMC2011/7/B7207

885 TGACGGCTGGAACTATACCGACAACTACATGACCGGTCGTACCAACGGCGTCGCAACCTA OmpK-35_YMC2011/8/B10311

*************** ********************************************

947 CCGTAACTCCGACTTCTTCGGTCTGGTTGACGGTCTGAGCTTCGCGCTGCAGTACCAGGG OmpK-35_YMC2013/7/B3993

945 CCGTAACTCCGACTTCTTCGGTCTGGTTGACGGTCTGAGCTTCGCGCTGCAGTACCAGGG OmpK-35_YMC2012/8/C631

945 CCGTAACTCCGACTTCTTCGGTCTGGTTGACGGTCTGAGCTTCGCGCTGCAGTACCAGGG OmpK-35_YMC2011/7/B774

940 CCGTAACTCCGACTTCTTCGGTCTGGTTGACGGTCTGAGCTTCGCGCTGCAGTACCAGGG OmpK-35_YMC2010/8/B2027

944 CCGTAACTCCGACTTCTTCGGTCTGGTTGACGGTCTGAGCTTCGCGCTGCAGTACCAGGG OmpK-35_YMC2011/11/B7578

945 CCGTAACTCTGACTTCTTCGGTCTGGTTGACGGTCTGAGCTTCGCGCTGCAGTACCAGGG OmpK-35_YMC2011/7/B7207

945 CCGTAACTCCGACTTCTTCGGTCTGGTTGACGGTCTGAGCTTCGCGCTGCAGTACCAGGG OmpK-35_YMC2011/8/B10311

********* **************************************************

1007 TAAAAA OmpK-35_YMC2013/7/B3993

1005 TAAAAA OmpK-35_YMC2012/8/C631

1005 TAAAAA OmpK-35_YMC2011/7/B774

1000 TAAAAA OmpK-35_YMC2010/8/B2027

1004 TAAAAA OmpK-35_YMC2011/11/B7578

1005 TAAAAA OmpK-35_YMC2011/7/B7207

1005 TAAAAA OmpK-35_YMC2011/8/B10311

******

Figure S3: Nucleotide alignment of OmpK 36 gene of panel strain: Multiple point mutations detected in YMC2011/7/B7207, YMC2011/8/B10311 and YMC2011/7/B774 (listed in table S3). OmpK 36 gene of YMC2012/8/C631 has been interrupted due to the IS element insertion (highlighted in yellow). Arrows indicate the start codon, RBS, -10, -35 and transcription regulators.

1 AATTAAAGTTGTGTAAAGAAGGGTAAAAAAAACCGGATGCGAGGCATCCGGTTGAAATAG OmpK-36_YMC2011/7/B7207

1 AATTAAAGTTGTGTAAAGAAGGGTAAAAAAAACCGGATGCGAGGCATCCGGTTGAAATAG OmpK-36_YMC2011/8/B10311

1 AATTAAAGTTGTGTAAAGAAGGGTAAAAAAAACCGGATGCGAGGCATCCGGTTGAAATAG OmpK-36_YMC2011/7/B774

1 AATTAAAGTTGTGTAAAGAAGGGTAAAAAAAACCGGATGCGAGGCATCCGGTTGAAATAG OmpK-36_YMC2012/8/C631

1 AATTAAAGTTGTGTAAAGAAGGGTAAAAAAAACCGGATGCGAGGCATCCGGTTGAAATAG OmpK-36_YMC2010/8/B2027

1 AATTAAAGTTGTGTAAAGAAGGGTAAAAAAAACCGGATGCGAGGCATCCGGTTGAAATAG OmpK-36_YMC2011/11/B7578

1 AATTAAAGTTGTGTAAAGAAGGGTAAAAAAAACCGGATGCGAGGCATCCGGTTGAAATAG OmpK-36_YMC2013/7/B3993

************************************************************

61 GGGTAAACAGACATTCAGAACTGAATGACGGTAATAAATAAAGTTAATGATGATAGCGAC OmpK-36_YMC2011/7/B7207

61 GGGTAAACAGACATTCAGAACTGAATGACGGTAATAAATAAAGTTAATGATGATAGCGAC OmpK-36_YMC2011/8/B10311

61 GGGTAAACAGACATTCAGAACTGAATGACGGTAATAAATAAAGTTAATGATGATAGCGAC OmpK-36_YMC2011/7/B774

61 GGGTAAACAGACATTCAGAACTGAATGACGGTAATAAATAAAGTTAATGATGATAGCGAC OmpK-36_YMC2012/8/C631

61 GGGTAAACAGACATTCAGAACTGAATGACGGTAATAAATAAAGTTAATGATGATAGCGAC OmpK-36_YMC2010/8/B2027

61 GGGTAAACAGACATTCAGAACTGAATGACGGTAATAAATAAAGTTAATGATGATAGCGAC OmpK-36_YMC2011/11/B7578

61 GGGTAAACAGACATTCAGAACTGAATGACGGTAATAAATAAAGTTAATGATGATAGCGAC OmpK-36_YMC2013/7/B3993

************************************************************

121 TCTTATTTTAGCCACCAATGATCATTTTGTTTTACCATTCAGTGCTATAGATATGCTGTC OmpK-36_YMC2011/7/B7207

121 TCTTATTTTAGCCACCAATGATCATTTTGTTTTACCATTCAGTGCTATAGATATGCTGTC OmpK-36_YMC2011/8/B10311

121 TCTTATTTTAGCCACCAATGATCATTTTGTTTTACCATTCAGTGCTATAGATATGCTGTC OmpK-36_YMC2011/7/B774

121 TCTTATTTTAGCCACCAATGATCATTTTGTTTTACCATTCAGTGCTATAGATATGTTGTT OmpK-36_YMC2012/8/C631

121 TCTTATTTTAGCCACCAATGATCATTTTGTTTTACCATTCAGTGCTATAGATATGTTGTT OmpK-36_YMC2010/8/B2027

121 TCTTATTTTAGCCACCAATGATCATTTTGTTTTACCATTCAGTGCTATAGATATGCTGTC OmpK-36_YMC2011/11/B7578

121 TCTTATTTTAGCCACCAATGATCATTTTGTTTTACCATTCAGTGCTATAGATATGCTGTC OmpK-36_YMC2013/7/B3993

******************************************************* ***

181 GTCTATCACTTTGATTATAAAAGGTTAAATATTTTTTTTGATGATTAGTGCGTATTTCCC OmpK-36_YMC2011/7/B7207

181 GTCTATCAATTTGATTATAAAAGGTTAAATATTTTTTTTGATGATTAGTGCGTATTTCCC OmpK-36_YMC2011/8/B10311

181 GTCTATCACTTTGATTATAAAAGGTTAAATATTTTTTTTGATGATTAGTGCGTATTTCCC OmpK-36_YMC2011/7/B774

181 GTCTATCAATTTGATTATAAAAGGTTAAATATTTTTTTTGATGATTAGTGCGTATTTCCC OmpK-36_YMC2012/8/C631

181 GTCTATCAATTTGATTATAAAAGGTTAAATATTTTTTTTGATGATTAGTGCGTATTTCCC OmpK-36_YMC2010/8/B2027

181 GTCTATCGCTTTGATTATAAAAGGTTAAATATTTTTTTTGATGATTAGTGCGTATTTCCC OmpK-36_YMC2011/11/B7578

181 GTCTATCGCTTTGATTATAAAAGGTTAAATATTTTTTTTGATGATTAGTGCGTATTTCCC OmpK-36_YMC2013/7/B3993

******* ***************************************************

IHF

241 TGACCATTTTGCGGTGAAAAAAGTTCCGCTGAATTTACAAATTGAAACATCTTGTGGGAA OmpK-36_YMC2011/7/B7207

241 TGACCATTTTGCGGTGAAAAAAGTTCCGCTGAATTTACAAATTGAAACATCTTGTGGGAA OmpK-36_YMC2011/8/B10311

241 TGACCATTTTGCGGTGAAAAAAGTTCCGCTGAATTTACAAATTGAAACATCTTGTGGGAA OmpK-36_YMC2011/7/B774

241 TGACCATTTTGCGGTAAAAAAAGTTCCGCTAAATTTACAAATTGAAACATCTTGTGGGAA OmpK-36_YMC2012/8/C631

241 TGACCATTTTGCGGTAAAAAAAGTTCCGCTAAATTTACAAATTGAAACATCTTGTGGGAA OmpK-36_YMC2010/8/B2027

241 TGACCATTTTGCGGTGAAAAAAGTTCCGCTAAATTTACAAATTGAAACATCTTGTGGGAA OmpK-36_YMC2011/11/B7578

241 TGACCATTTTGCGGTGAAAAAAGTTCCGCTAAATTTACAAATTGAAACATCTTGTGGGAA OmpK-36_YMC2013/7/B3993

*************** ************** *****************************

C1

301 CTTTGAAACATCTTAGAAGTTTTAGTATCATATTCTTGTTGGATTATTCTGCATTTTGCA OmpK-36_YMC2011/7/B7207

301 CTTTGAAACATCTTAGAAGTTTTAGTATCATATTCTTGTTGGATTATTCTGCATTTTGCA OmpK-36_YMC2011/8/B10311

301 CTTTGAAACATCTTAGAAGTTTTAGTATCATATTCTTGTTGGATTATTCTGCATTTTGCA OmpK-36_YMC2011/7/B774

301 CTTTGAAACATCTTAGAAGTTTTAGTATCATATTCTTGTTGGATTATTCTGCATTTTGCA OmpK-36_YMC2012/8/C631

301 CTTTGAAACATCTTAGAAGTTTTAGTATCATATTCTTGTTGGATTATTCTGCATTTTGCA OmpK-36_YMC2010/8/B2027

301 CTTTGAAACATCTTAGAAGTTTTAGTATCATATTCTTGTTGGATTATTCTGCATTTTGCA OmpK-36_YMC2010/8/B2027

301 CTTTGAAACATCTTAGAAGTTTTAGTATCATATTCTTGTTGGATTATTCTGCATTTTGCA OmpK-36_YMC2013/7/B3993

************************************************************

C2 C3 -35

361 GCACAATGAAATAGCCGACTGATTAGAAGGGTAATCAGTAAGCAGTGGCATAATAAAAGG OmpK-36_YMC2011/7/B7207

361 GCACAATGAAATAGCCGACTGATTAGAAGGGTAATCAGTAAGCAGTGGCATAATAAAAGG OmpK-36_YMC2011/8/B10311

361 GCACAATGAAATAGCCGACTGATTAGAAGGGTAATCAGTAAGCAGTGGCATAATAAAAGG OmpK-36_YMC2011/7/B774

361 GCACAATGAAATAGCCGACTGATTAGAAGGGTAATCAGTAAGCAGTGGCATAATAAAAGG OmpK-36_YMC2012/8/C631

361 GCACAATGAAATAGCCGACTGATTAGAAGGGTAATCAGTAAGCAGTGGCATAATAAAAGG OmpK-36_YMC2010/8/B2027

361 GCACAATGAAATAGCCGACTGATTAGAAGGGTAATCAGTAAGCAGTGGCATAATAAAAGG OmpK-36_YMC2011/11/B7578

361 GCACTATGAAATAGCCGACTGATTAGAAGGGTAATCAGTAAGCAGTGGCATAATAAAAGG OmpK-36_YMC2013/7/B3993

**** *******************************************************

-10

421 CATATAACAAACAGAGGGTTAATAACATGAAAGTTAAAGTACTGTCCCTCCTGGTACCGG OmpK-36_YMC2011/7/B7207

421 CATATAACAAACAGAGGGTTAATAACATGAAAGTTAAAGTACTGTCCCTCCTGGTACCGG OmpK-36_YMC2011/8/B10311

421 CATATAACAAACAGAGGGTTAATAACATGAAAGTTAAAGTACTGTCCCTCTTGGTACCGG OmpK-36_YMC2011/7/B774

421 CATATAACAAACAGAGGGTTAATAACATGAAAGTTAAAGTACTGTCCCTCCTGGTACCGG OmpK-36_YMC2012/8/C631

421 CATATAACAAACAGAGGGTTAATAACATGAAAGTTAAAGTACTGTCCCTCCTGGTACCGG OmpK-36_YMC2010/8/B2027

421 CATATAACAAACAGAGGGTTAATAACATGAAAGTTAAAGTACTGTCCCTCCTGGTACCGG OmpK-36_YMC2011/11/B7578

421 CATATAACAAACAGAGGGTTAATAACATGAAAGTTAAAGTACTGTCCCTCCTGGTACCGG OmpK-36_YMC2013/7/B3993

************************************************** *********

RBS Start

481 CTCTGCTGGTAGCAGGCGCAGCAAATGCGGCTGAAATTTATAACAAAGACGGCAACAAAT OmpK-36_YMC2011/7/B7207

481 CTCTGCTGGTAGCAGGCGCAGCAAATGCGGCTGAAATTTATAACAAAGACGGCAACAAAT OmpK-36_YMC2011/8/B10311

481 CTCTGCTGGTAGCAGGCGCAGCAAATGCGGCTGAAATTTATAACAAAGACGGCAACAAAT OmpK-36_YMC2011/7/B774

481 CTCTGCTGGTAGCAGGCGCAGCAAATGCGGCTGAAATTTATAACAAAGACGGCAACAAAT OmpK-36_YMC2012/8/C631

481 CTCTGCTGGTAGCAGGCGCAGCAAATGCGGCTGAAATTTATAACAAAGACGGCAACAAAT OmpK-36_YMC2010/8/B2027

481 CTCTGCTGGTAGCAGGCGCAGCAAATGCGGCTGAAATTTATAACAAAGACGGCAACAAAT OmpK-36_YMC2011/11/B7578

481 CTCTGCTGGTAGCAGGCGCAGCAAATGCGGCTGAAATTTATAACAAAGACGGCAACAAAT OmpK-36_YMC2013/7/B3993

************************************************************

541 TAGACCTGTACGGTAAAATTGACGGTCTGCACTACTTCTCTGACGACAAGAG-------- OmpK-36_YMC2011/7/B7207

541 TAGACCTGTACGGTAAAATTGACGGTCTGCACTACTTCTCTGACGACAAGAG-------- OmpK-36_YMC2011/8/B10311

541 TAGACCTGTATGGTAAAATTGACGGTCTGCACTACTTCTCTGACGACAAGAG-------- OmpK-36_YMC2011/7/B774

541 TAGACCTGTACGGTAAAATTGACGGTCTGCACTACTTCTCTGACGACAAGAGCGTCGACG OmpK-36_YMC2012/8/C631

541 TAGACCTGTACGGTAAAATTGACGGTCTGCACTACTTCTCTGACGACAAGAG-------- OmpK-36_YMC2010/8/B2027

541 TAGACCTGTACGGTAAAATTGACGGTCTGCACTACTTCTCTGACGACAAGAG-------- OmpK-36_YMC2011/11/B7578

541 TAGACCTGTACGGTAAAATTGACGGTCTGCACTACTTCTCTGACGACAAGAG-------- OmpK-36_YMC2013/7/B3993

********** *****************************************

593 ------------------------CGTCGACGGCGACCAGACCTACATGCGTGTAGGCGT OmpK-36_YMC2011/7/B7207

593 ------------------------CGTCGACGGCGACCAGACCTACATGCGTGTAGGCGT OmpK-36_YMC2011/8/B10311

593 ------------------------CGTCGACGGCGACCAGACCTACATGCGTGTAGGCGT OmpK-36_YMC2011/7/B774

601 GCGACCATTCTCTGACGACAAGAGCGTCGACGGCGACCAGACCTACATGCGTGTAGGCGT OmpK-36_YMC2012/8/C631

593 ------------------------CGTCGACGGCGACCAGACCTACATGCGTGTAGGCGT OmpK-36_YMC2010/8/B2027

593 ------------------------CGTCGACGGCGACCAGACCTACATGCGTGTAGGCGT OmpK-36_YMC2011/11/B7578

593 ------------------------CGTCGACGGCGACCAGACCTACATGCGTGTAGGCGT OmpK-36_YMC2013/7/B3993

************************************

629 GAAAGGCGAAACCCAGATCAACGACCAGCTGACCGGTTACGGCCAGTGGGAATACAACGT OmpK-36_YMC2011/7/B7207

629 GAAAGGCGAAACCCAGATCAACGACCAGCTGACCGGTTACGGCCAGTGGGAATACAACGT OmpK-36_YMC2011/8/B10311

629 GAAAGGCGAAACCCAGATCAACGACCAGCTGACCGGTTACGGCCAGTGGGAATACAACGT OmpK-36_YMC2011/7/B774

661 GAAAGGCGAAACCCAGATCAACGACCAGCTGACCGGTTACGGCCAGTGGGAATACAACGT OmpK-36_YMC2012/8/C631

629 GAAAGGCGAAACCCAGATCAACGACCAGCTGACCGGTTACGGCCAGTGGGAATACAACGT OmpK-36_YMC2010/8/B2027

629 GAAAGGCGAAACCCAGATCAACGACCAGCTGACCGGTTACGGCCAGTGGGAATACAACGT OmpK-36_YMC2011/11/B7578

629 GAAAGGCGAAACCCAGATCAACGACCAGCTGACCGGTTACGGCCAGTGGGAATACAACGT OmpK-36_YMC2013/7/B3993

************************************************************

689 TCAGGCGAACAACGTTGAAGGTACTGACAAGCAGTCCTGGACTCGTCTGGCATTCGCGGG OmpK-36_YMC2011/7/B7207

689 TCAGGCGAACAACGTTGAAGGTACTGACAAGTAGTCCTGGACTCGTCTGGCATTCGCGGG OmpK-36_YMC2011/8/B10311

689 TCAGGCGAACAACACTGAAAGCTCCAGCGATCAGGCATGGACTCGTCTGGCATTCGCAGG OmpK-36_YMC2011/7/B774

721 TCAGGCGAACAACACTGAAAGCTCCAGCGATCAGGCATGGACTCGTCTGGCATTCGCAGG OmpK-36_YMC2012/8/C631

689 TCAGGCGAACAACACTGAAAGCTCCAGCGATCAGGCATGGACTCGTCTGGCATTCGCAGG OmpK-36_YMC2010/8/B2027

689 TCAGGCGAACAACACTGAAAGCTCCAGCGATCAGGCATGGACTCGTCTGGCATTCGCAGG OmpK-36_YMC2011/11/B7578

689 TCAGGCGAACAACACTGAAAGCTCCAGCGATCAGGCATGGACTCGTCTGGCATTCGCAGG OmpK-36_YMC2013/7/B3993

************* **** * * * * ** * ******************** **

789 TCTGAAATTCGGCGACGCGGGTTCTTTCGACTACGGTCGTAACTACGGCGTTGTTTACGA OmpK-36_YMC2011/7/B7207

749 TCTGAAATTCGGCGACGCGGGTTCTTTCGACTACGGTCGTAACTACGGCGTTGTTTACGA OmpK-36_YMC2011/8/B10311

749 CCTGAAATTTGGCGACGCGGGCTCTTTCGACTACGGTCGTAACTACGGCGTAGTATACGA OmpK-36_YMC2011/7/B774

781 CCTGAAATTTGGCGACGCGGGCTCTTTCGACTACGGTCGTAACTACGGCGTAGTATACGA OmpK-36_YMC2012/8/C631

749 CCTGAAATTTGGCGACGCGGGCTCTTTCGACTACGGTCGTAACTACGGCGTAGTATACGA OmpK-36_YMC2010/8/B2027

749 CCTGAAATTTGGCGACGCGGGCTCTTTCGACTACGGTCGTAACTACGGCGTAGTATACGA OmpK-36_YMC2011/11/B7578

749 CCTGAAATTTGGCGACGCGGGCTCTTTCGACTACGGTCGTAACTACGGCGTAGTATACGA OmpK-36_YMC2013/7/B3993

******** *********** ***************************** ** *****

809 CGTGACTTCCTGGACCGACGTTCTGCCGGAATTCGGCGGCGACACCTACGGTTCCGACAA OmpK-36_YMC2011/7/B7207

809 CGTGACTTCCTGGACCGACGTTCTGCCGGAATTCGGCGGCGACACCTACGGTTCCGACAA OmpK-36_YMC2011/8/B10311

809 CGTAACGTCCTGGACCGACGTTCTGCCGGAATTCGGCGGCGACACCTACGGTTCTGACAA OmpK-36_YMC2011/7/B774

841 CGTAACGTCCTGGACCGACGTTCTGCCGGAATTCGGCGGCGACACCTACGGTTCTGACAA OmpK-36_YMC2012/8/C631

809 CGTAACGTCCTGGACCGACGTTCTGCCGGAATTCGGCGGCGACACCTACGGTTCTGACAA OmpK-36_YMC2010/8/B2027

809 CGTAACGTCCTGGACCGACGTTCTGCCGGAATTCGGCGGCGACACCTACGGTTCTGACAA OmpK-36_YMC2011/11/B7578

809 CGTAACGTCCTGGACCGACGTTCTGCCGGAATTCGGCGGCGACACCTACGGTTCTGACAA OmpK-36_YMC2013/7/B3993

*** ** *********************************************** *****

869 CTTCCTGCAGTCCCGTGCTAACGGCGTTGCAACCTATCGTAACTCTGACTTCTTCGGTCT OmpK-36_YMC2011/7/B7207

869 CTTCCTGCAGTCCCGTGCTAACGGCGTTGCAACCTATCGTAACTCTGACTTCTTCGGTCT OmpK-36_YMC2011/8/B10311

869 CTTCCTGCAGTCCCGTGCTAACGGCGTTGCAACCTACCGTAACTCTGATTTCTTCGGTCT OmpK-36_YMC2011/7/B774

901 CTTCCTGCAGTCCCGTGCTAACGGCGTTGCAACCTACCGTAACTCTGATTTCTTCGGTCT OmpK-36_YMC2012/8/C631

869 CTTCCTGCAGTCCCGTGCTAACGGCGTTGCAACCTACCGTAACTCTGATTTCTTCGGTCT OmpK-36_YMC2010/8/B2027

869 CTTCCTGCAGTCCCGTGCTAACGGCGTTGCAACCTACCGTAACTCTGATTTCTTCGGTCT OmpK-36_YMC2011/11/B7578

869 CTTCCTGCAGTCCCGTGCTAACGGCGTTGCAACCTACCGTAACTCTGATTTCTTCGGTCT OmpK-36_YMC2013/7/B3993

************************************ *********** ***********

929 GGTTGACGGCCTGAACTTTGCCCTGCAGTACCAAGGTAAAAACGGCAGCGTCAGCGGCGA OmpK-36_YMC2011/7/B7207

929 GGTTGACGGCCTGAACTTTGCCCTGCAGTACCAAGGTAAAAACGGCAGCGTCAGCGGCGA OmpK-36_YMC2011/8/B10311

929 GGTTGACGGCCTGAACTTTGCTCTGCAGTATCAGGGTAAAAACGGCAGCGTCAGCGGCGA OmpK-36_YMC2011/7/B774

961 GGTTGACGGCCTGAACTTTGCTCTGCAGTATCAGGGTAAAAACGGCAGCGTCAGCGGCGA OmpK-36_YMC2012/8/C631

929 GGTTGACGGCCTGAACTTTGCTCTGCAGTATCAGGGTAAAAACGGCAGCGTCAGCGGCGA OmpK-36_YMC2010/8/B2027

929 GGTTGACGGCCTGAACTTTGCTCTGCAGTATCAGGGTAAAAACGGCAGCGTCAGCGGCGA OmpK-36_YMC2011/11/B7578

929 GGTTGACGGCCTGAACTTTGCTCTGCAGTATCAGGGTAAAAACGGCAGCGTCAGCGGCGA OmpK-36_YMC2013/7/B3993

********************* ******** ** **************************

989 AGACATGA---------CCAACAACGGTCGTGGCGCTCAGAAACAGAACGGCGACGGCTT OmpK-36_YMC2011/7/B7207

989 AGACATGA---------CCAACAACGGTCGTGGCGCTCAGAAACAGAACGGCGACGGCTT OmpK-36_YMC2011/8/B10311

989 AGGCGCGA---------CCAACAACGGTCGTGGTTGGAGCAAACAGAACGGCGACGGCTT OmpK-36_YMC2011/7/B774

1021 AGGCGCTCTGTCTCCTACCAACAACGGTCGTACCGCCTTGAAACAGAACGGCGACGGTTA OmpK-36_YMC2012/8/C631

989 AGGCGCTCTGTCTCCTACCAACAACGGTCGTACCGCCTTGAAACAGAACGGCGACGGTTA OmpK-36_YMC2010/8/B2027

989 AGGCGCTCTGTCTCCTACCAACAACGGTCGTACCGCCTTGAAACAGAACGGCGACGGTTA OmpK-36_YMC2011/11/B7578

989 AGGCGCTCTGTCTCCTACCAACAACGGTCGTACCGCCTTGAAACAGAACGGCGACGGTTA OmpK-36_YMC2013/7/B3993

** * ************** ***************** *

1040 CGGCACCTCCGTAACTTATGATATCTGGGACGGCATCAGCGCTGGTTTCGCTTACTCCCA OmpK-36_YMC2011/7/B7207

1040 CGGCACCTCCGTAACTTATGATATCTGGGACGGCATCAGCGCTGGTTTCGCTTACTCCCA OmpK-36_YMC2011/8/B10311

1040 CGGCACCTCTCTGACCTACGATATTTGGGATGGCATCAGCGCTGGTTTCGCGTACTCGCA OmpK-36_YMC2011/7/B774

1081 CGGTACTTCTCTGACCTATGACATCTATGATGGCATCAGCGCTGGTTTCGCATACTCTAA OmpK-36_YMC2012/8/C631

1049 CGGTACTTCTCTGACCTATGACATCTATGATGGCATCAGCGCTGGTTTCGCATACTCTAA OmpK-36_YMC2010/8/B2027

1049 CGGTACTTCTCTGACCTATGACATCTATGATGGCATCAGCGCTGGTTTCGCATACTCTAA OmpK-36_YMC2011/11/B7578

1049 CGGTACTTCTCTGACCTATGACATCTATGATGGCATCAGCGCTGGTTTCGCATACTCTAA OmpK-36_YMC2013/7/B3993

*** ** ** * ** ** ** ** * ** ******************** ***** *

1100 CTCTAAACGTACTGATGACCAGAAC---AACCTGGTACTGGGTCGTGGTGACGACGCTGA OmpK-36_YMC2011/7/B7207

1100 CTCTAAACGTACTGATGACCAGAAC---AACCTGGTACTGGGTCGTGGTGACGACGCTGA OmpK-36_YMC2011/8/B10311

1100 CTCCAAACGTACCGACGAGCAGAATAGTGTTCCGGCACTGGGTCGTGGCGACAACGCTGA OmpK-36_YMC2011/7/B774

1141 CTCCAAACGTCTTGGCGACCAGAACAGCAAGCTGGCACTGGGTCGTGGCGACAACGCTGA OmpK-36_YMC2012/8/C631

1109 CTCCAAACGTCTTGGCGACCAGAACAGCAAGCTGGCACTGGGTCGTGGCGACAACGCTGA OmpK-36_YMC2010/8/B2027

1109 CTCCAAACGTCTTGGCGACCAGAACAGCAAGCTGGCACTGGGTCGTGGCGACAACGCTGA OmpK-36_YMC2011/11/B7578

1109 CTCCAAACGTCTTGGCGACCAGAACAGCAAGCTGGCACTGGGTCGTGGCGACAACGCTGA OmpK-36_YMC2013/7/B3993

*** ****** * ** ***** * ** ************ **********

1157 AACCTATACCGGTGGTCTGAAATACGACGCCAACAACATCTACCTGGCCTCTCAGTACAC OmpK-36_YMC2011/7/B7207

1157 AACCTATACCGGTGGTCTGAAATACGACGCCAACAACATCTACCTGGCCTCTCAGTACAC OmpK-36_YMC2011/8/B10311

1160 AACCTACACCGGTGGTCTGAAATACGACGCCAACAACATCTACCTGGCCTCTCAGTACAC OmpK-36_YMC2011/7/B774

1201 AACCTACACCGGCGGTCTGAAATACGACGCGAACAACATCTACCTGGCCACTCAGTACAC OmpK-36_YMC2012/8/C631

1169 AACCTACACCGGCGGTCTGAAATACGACGCGAACAACATCTACCTGGCCACTCAGTACAC OmpK-36_YMC2010/8/B2027

1169 AACCTACACCGGCGGTCTGAAATACGACGCGAACAACATCTACCTGGCCACTCAGTACAC OmpK-36_YMC2011/11/B7578

1169 AACCTACACCGGCGGTCTGAAATACGACGCGAACAACATCTACCTGGCCACTCAGTACAC OmpK-36_YMC2013/7/B3993

****** ***** ***************** ****************** **********

1217 CCAGACCTACAACGCAACTCGCGCCGGTTCCCTGGGCTTTGCAAACAAAGCGCAGAACTT OmpK-36_YMC2011/7/B7207

1217 CCAGACCTACAACGCAACTCGCGCCGGTTCCCTGGGCTTTGCAAACAAAGCGCAGAACTT OmpK-36_YMC2011/8/B10311

1220 CCAGACCTACAACGCAACTCGCGCCGGTTCCCTGGGCTTTGCAAACAAAGCGCAGAACTT OmpK-36_YMC2011/7/B774

1261 CCAGACCTACAACGCGACCCGCGCCGGTTCCCTGGGCTTTGCTAACAAAGCGCAGAACTT OmpK-36_YMC2012/8/C631

1229 CCAGACCTACAACGCGACCCGCGCCGGTTCCCTGGGCTTTGCTAACAAAGCGCAGAACTT OmpK-36_YMC2010/8/B2027

1229 CCAGACCTACAACGCGACCCGCGCCGGTTCCCTGGGCTTTGCTAACAAAGCGCAGAACTT OmpK-36_YMC2011/11/B7578

1229 CCAGACCTACAACGCGACCCGCGCCGGTTCCCTGGGCTTTGCTAACAAAGCGCAGAACTT OmpK-36_YMC2013/7/B3993

*************** ** *********************** *****************

1277 CGAAGTGGTTGCTCAGTACCAGTTCGACTTCGGTCTGCGTCCGTCTGTGGCTTACCTGCA OmpK-36_YMC2011/7/B7207

1277 CGAAGTGGTTGCTCAGTACCAGTTCGACTTCGGTCTGCGTCCGTCTGTGGCTTACCTGCA OmpK-36_YMC2011/8/B10311

1280 CGAAGTGGTTGCTCAGTACCAGTTCGACTTCGGTCTGCGTCCGTCTGTGGCTTACCTGCA OmpK-36_YMC2011/7/B774

1321 CGAAGTGGTTGCTCAGTACCAGTTCGACTTCGGTCTGCGTCCGTCCGTGGCTTACCTGCA OmpK-36_YMC2012/8/C631

1289 CGAAGTGGTTGCTCAGTACCAGTTCGACTTCGGTCTGCGTCCGTCCGTGGCTTACCTGCA OmpK-36_YMC2010/8/B2027

1289 CGAAGTGGTTGCTCAGTACCAGTTCGACTTCGGTCTGCGTCCGTCCGTGGCTTACCTGCA OmpK-36_YMC2011/11/B7578

1289 CGAAGTGGTTGCTCAGTACCAGTTCGACTTCGGTCTGCGTCCGTCCGTGGCTTACCTGCA OmpK-36_YMC2013/7/B3993

********************************************* **************

1337 GTCTAAAGGTAAGGATCTGGAGCGCGGCTACGGCGACCAGGACATCCTGAAATATGTTGA OmpK-36_YMC2011/7/B7207

1337 GTCTAAAGGTAAGGATCTGGAGCGCGGCTACGGCGACCAGGACATCCTGAAATATGTTGA OmpK-36_YMC2011/8/B10311

1340 GTCTAAAGGTAAGGATCTGGAGCGCGGCTACGGCGACCAGGACATCCTGAAATATGTTGA OmpK-36_YMC2011/7/B774

1438 GTCTAAAGGTAAGGATCTGGAAG---GCTACGGCGACCAGGACATCCTGAAATATGTTGA OmpK-36_YMC2012/8/C631

1406 GTCTAAAGGTAAGGATCTGGAAG---GCTACGGCGACCAGGACATCCTGAAATATGTTGA OmpK-36_YMC2010/8/B2027

1406 GTCTAAAGGTAAGGATCTGGAAG---GCTACGGCGACTAGGACATCCTGAAATATGTTGA OmpK-36_YMC2011/11/B7578

1406 GTCTAAAGGTAAGGATCTGGAAG---GCTACGGCGACCAGGACATCCTGAAATATGTTGA OmpK-36_YMC2013/7/B3993

********************* *********** **********************

1397 CGTTGGCGCGACCTACTACTTCAACAAAAACATGTCCACCTATGTTGACTACAAAATCAA OmpK-36_YMC2011/7/B7207

1397 CGTTGGCGCGACCTACTACTTCAACAAAAACATGTCCACCTATGTTGACTACAAAATCAA OmpK-36_YMC2011/8/B10311

1400 CGTTGGCGCGACCTACTACTTCAACAAAAACATGTCCACCTATGTTGACTACAAAATCAA OmpK-36_YMC2011/7/B774

1438 CGTTGGCGCGACCTACTACTTCAACAAAAACATGTCCACCTATGTTGACTACAAAATCAA OmpK-36_YMC2012/8/C631

1406 CGTTGGCGCGACCTACTACTTCAACAAAAACATGTCCACCTATGTTGACTACAAAATCAA OmpK-36_YMC2010/8/B2027

1406 CGTTGGCGCGACCTACTACTTCAACAAAAACATGTCCACCTATGTTGACTACAAAATCAA OmpK-36_YMC2011/11/B7578

1406 CGTTGGCGCGACCTACTACTTCAACAAAAACATGTCCACCTATGTTGACTACAAAATCAA OmpK-36_YMC2013/7/B3993

************************************************************

1457 CCTGCTGGACGACAACAGCTTCACCCGCAACGCCGGTATCTCTACCGACGACGTGGTTGC OmpK-36_YMC2011/7/B7207

1457 CCTGCTGGACGACAACAGCTTCACCCGCAACGCCGGTATCTCTACCGACGACGTGGTTGC OmpK-36_YMC2011/8/B10311

1460 CCTGCTGGACGACAACAGCTTCACCCGCAACGCCGGTATCTCTACCGACGACGTGGTTGC OmpK-36_YMC2011/7/B774

1498 CCTGCTGGACGACAACAGCTTCACCCGCAACGCCGGTATCTCTACCGACGACGTGGTTGC OmpK-36_YMC2012/8/C631

1466 CCTGCTGGACGACAACAGCTTCACCCGCAACGCCGGTATCTCTACCGACGACGTGGTTGC OmpK-36_YMC2010/8/B2027

1466 CCTGCTGGACGACAATAGCTTCACCCACAACGCCGGTATCTCTACCGACGACGTGGTTGC OmpK-36_YMC2011/11/B7578

1466 CCTGCTGGACGACAATAGCTTCACCCACAACGCCGGTATCTCTACCGACGACGTGGTTGC OmpK-36_YMC2013/7/B3993

*************** ********** *********************************

1517 ACTGGGCCTGGTTTACCAGTTCTAA OmpK-36_YMC2011/7/B7207

1417 ACTGGGCCTGGTTTACCAGTTCTAA OmpK-36_YMC2011/8/B10311

1520 ACTGGGCCTGGTTTACCAGTTCTAA OmpK-36_YMC2011/7/B774

1558 ACTGGGCCTGGTTTACCAGTTCTAA OmpK-36_YMC2012/8/C631

1526 ACTGGGCCTGGTTTACCAGTTCTAA OmpK-36_YMC2010/8/B2027

1526 ACTGGGCCTGGTTTACCAGTTCTAA OmpK-36_YMC2011/11/B7578

1526 ACTGGGCCTGGTTTACCAGTTCTAA OmpK-36_YMC2013/7/B3993

*************************

Figure S4: Amino acid alignment of SHV-11, SHV-12, SHV-187 and SHV-158 of Panel strains along with the reference sequences: Clustal 0 (1.2.3) multiple sequence alignment of SHV genes. SHV-12_AJ920369, SHV-187_LN515533, SHV-158_JX121125 and SHV-11_X98101 are the reference sequences. Polymorphism are highlighted in grey.

1 MRYIRLCIISLLATLPLAVHASPQPLEQIKQSESQLSGRVGMIEMDLASGRTLTAWRADE SHV-12_AJ920369

1 MRYIRLCIISLLATLPLAVHASPQPLEQIKQSESQLSGRVGMIEMDLASGRTLTAWRADE SHV-12_YMC2011/11/B7578

1 MRYIRLCIISLLATLPLAVHASPQPLEQIKQSESQLSGRVGMIEMDLASGRTLTAWRADE SHV-12_YMC2013/7/B3993_1

1 MRYIRLCIISLLATLPLAVHASPQPLEQIKQSESQLSGRVGMIEMDLASGRTLTAWRADE SHV-12_YMC2013/7/B3993_2

1 KRYIRLCIISLLATLPLAVHASPQPLEQIKLSESQLSGRVGMIEMDLASGRTLTAWRADE SHV-187_LN515533

1 KRYIRLCIISLLATLPLAVHASPQPLEQIKLSESQLSGRVGMIEMDLASGRTLTAWRADE SHV-187_YMC2011/7/B7207

1 MRYIRLCIISLLATLPLAVHASPQPLEQIKQSESQLSGRVGMIEMDLASGRTLAAWRADE SHV-158_JX121125

1 MRYIRLCIISLLATLPLAVHASPQPLEQIKQSESQLSGRVGMIEMDLASGRTLAAWRADE SHV-158_YMC2011/11/B7578

1 MRYIRLCIISLLATLPLAVHASPQPLEQIKQSESQLSGRVGMIEMDLASGRTLTAWRADE SHV-11_X98101

1 MRYIRLCIISLLATLPLAVHASPQPLEQIKQSESQLSGRVGMIEMDLASGRTLTAWRADE SHV-11_YMC2011/7/B774

1 MRYIRLCIISLLATLPLAVHASPQPLEQIKQSESQLSGRVGMIEMDLASGRTLTAWRADE SHV-11_YMC2011/8/B10311

1 MRYIRLCIISLLATLPLAVHASPQPLEQIKQSESQLSGRVGMIEMDLASGRTLTAWRADE SHV-11_YMC2010/8/B2027

***************************** **********************:******

61 RFPMMSTFKVVLCGAVLARVDAGDEQLERKIHYRQQDLVDYSPVSEKHLADGMTVGELCA SHV-12_AJ920369

61 RFPMMSTFKVVLCGAVLARVDAGDEQLERKIHYRQQDLVDYSPVSEKHLADGMTVGELCA SHV-12_YMC2011/11/B7578

61 RFPMMSTFKVVLCGAVLARVDAGDEQLERKIHYRQQDLVDYSPVSEKHLADGMTVGELCA SHV-12_YMC2013/7/B3993_1

61 RFPMMSTFKVVLCGAVLARVDAGDEQLERKIHYRQQDLVDYSPVSEKHLADGMTVGELCA SHV-12_YMC2013/7/B3993_2

61 RFPMMSTFKVVLCGAVLARVDAGDEQLERKIHYRQQDLVDYSPVSEKHLADGMTVGELCA SHV-187_LN515533

61 RFPMMSTFKVVLCGAVLARVDAGDEQLERKIHYRQQDLVDYSPVSEKHLADGMTVGELCA SHV-187_YMC2011/7/B7207

61 RFPMMSTFKVVLCGAVLARVDAGDEQLERKIHYRQQDLVDYSPVSEKHLADGMTVGELCA SHV-158_JX121125

61 RFPMMSTFKVVLCGAVLARVDAGDEQLERKIHYRQQDLVDYSPVSEKHLADGMTVGELCA SHV-158_YMC2011/11/B7578

61 RFPMMSTFKVVLCGAVLARVDAGDEQLERKIHYRQQDLVDYSPVSEKHLADGMTVGELCA SHV-11_X98101

61 RFPMMSTFKVVLCGAVLARVDAGDEQLERKIHYRQQDLVDYSPVSEKHLADGMTVGELCA SHV-11_YMC2011/7/B774

61 RFPMMSTFKVVLCGAVLARVDAGDEQLERKIHYRQQDLVDYSPVSEKHLADGMTVGELCA SHV-11_YMC2011/8/B10311

61 RFPMMSTFKVVLCGAVLARVDAGDEQLERKIHYRQQDLVDYSPVSEKHLADGMTVGELCA SHV-11_YMC2010/8/B2027

************************************************************

121 AAITMSDNSAANLLLATVGGPAGLTAFLRQIGDNVTRLDRWETELNEALPGDARDTTTPA SHV-12_AJ920369 0

121 AAITMSDNSAANLLLATVGGPAGLTAFLRQIGDNVTRLDRWETELNEALPGDARDTTTPA SHV-12_YMC2011/11/B7578

121 AAITMSDNSAANLLLATVGGPAGLTAFLRQIGDNVTRLDRWETELNEALPGDARDTTTPA SHV-12_YMC2013/7/B3993_1

121 AAITMSDNSAANLLLATVGGPAGLTAFLRQIGDNVTRLDRWETELNEALPGDARDTTTPA SHV-12_YMC2013/7/B3993_2

121 AAITMSDNSAANLLLATVGGPAGLTAFLRQIGDNVTRLDRWETELNEALPGDARDTTTPA SHV-187_LN515533

121 AAITMSDNSAANLLLATVGGPAGLTAFLRQIGDNVTRLDRWETELNEALPGDARDTTTPA SHV-187_YMC2011/7/B7207

121 AAITMSDNSAANLLLATVGGPAGLTAFLRQIGDNVTRLDRWETELNEALPGDARDTTTPA SHV-158_JX121125

121 AAITMSDNSAANLLLATVGGPAGLTAFLRQIGDNVTRLDRWETELNEALPGDARDTTTPA SHV-158_YMC2011/11/B7578

121 AAITMSDNSAANLLLATVGGPAGLTAFLRQIGDNVTRLDRWETELNEALPGDARDTTTPA SHV-11_X98101

121 AAITMSDNSAANLLLATVGGPAGLTAFLRQIGDNVTRLDRWETELNEALPGDARDTTTPA SHV-11_YMC2011/7/B774

121 AAITMSDNSAANLLLATVGGPAGLTAFLRQIGDNVTRLDRWETELNEALPGDARDTTTPA SHV-11_YMC2011/8/B10311

121 AAITMSDNSAANLLLATVGGPAGLTAFLRQIGDNVTRLDRWETELNEALPGDARDTTTPA SHV-11_YMC2010/8/B2027

************************************************************

181 SMAATLRKLLTSQRLSARSQRQLLQWMVDDRVAGPLIRSVLPAGWFIADKTGASKRGARG SHV-12_AJ920369

181 SMAATLRKLLTSQRLSARSQRQLLQWMVDDRVAGPLIRSVLPAGWFIADKTGASKRGARG SHV-12_YMC2011/11/B7578

181 SMAATLRKLLTSQRLSARSQRQLLQWMVDDRVAGPLIRSVLPAGWFIADKTGASKRGARG SHV-12_YMC2013/7/B3993_1

181 SMAATLRKLLTSQRLSARSQRQLLQWMVDDRVAGPLIRSVLPAGWFIADKTGASKRGARG SHV-12_YMC2013/7/B3993_2

181 SMAATLRKLLTSQRLSARSQRQLLQWMVDDRVAGPLIRSVLPAGWFIADKTGAGERGARG SHV-187_LN515533

181 SMAATLRKLLTSQRLSARSQRQLLQWMVDDRVAGPLIRSVLPAGWFIADKTGAGERGARG SHV-187_YMC2011/7/B7207

181 SMAATLRKLLTSQRLSARSQRQLLQWMVDDRVAGPLIRSVLPAGWFIADKTGAGERGARG SHV-158_JX121125

181 SMAATLRKLLTSQRLSARSQRQLLQWMVDDRVAGPLIRSVLPAGWFIADKTGAGERGARG 158_YMC2011/11/B7578

181 SMAATLRKLLTSQRLSARSQRQLLQWMVDDRVAGPLIRSVLPAGWFIADKTGAGERGARG SHV-11_X98101

181 SMAATLRKLLTSQRLSARSQRQLLQWMVDDRVAGPLIRSVLPAGWFIADKTGAGERGARG SHV-11_YMC2011/7/B774

181 SMAATLRKLLTSQRLSARSQRQLLQWMVDDRVAGPLIRSVLPAGWFIADKTGAGERGARG SHV-11_YMC2011/8/B10311

181 SMAATLRKLLTSQRLSARSQRQLLQWMVDDRVAGPLIRSVLPAGWFIADKTGAGERGARG SHV-11_YMC2010/8/B2027

*****************************************************.:*****

241 IVALLGPNNKAERIVVIYLRDTPASMAERNQQIAGIGAALIEHWQR SHV-12_AJ920369

241 IVALLGPNNKAERIVVIYLRDTPASMAERNQQIAGIGAALIEHWQR SHV-12_YMC2011/11/B7578

241 IVALLGPNNKAERIVVIYLRDTPASMAERNQQIAGIGAALIEHWQR SHV-12_YMC2013/7/B3993_1

241 IVALLGPNNKAERIVVIYLRDTPASMAERNQQIAGIGAALIEHWQR SHV-12_YMC2013/7/B3993_2

241 IVALLGPNNKAERIVVIYLRDTPASMAERNQQIAGIGAALIEHWQR SHV-187_LN515533

241 IVALLGPNNKAERIVVIYLRDTPASMAERNQQIAGIGAALIEHWQR SHV-187_YMC2011/7/B7207

241 IVALLGPNNKAERIVVIYLRDTPASMAERNQQIAGIGAALIEHWQR SHV-158_JX121125

241 IVALLGPNNKAERIVVIYLRDTPASMAERNQQIAGIGAALIEHWQR SHV-158_YMC2011/11/B7578

241 IVALLGPNNKAERIVVIYLRDTPASMAERNQQIAGIGAALIEHWQR SHV-11_X98101

241 IVALLGPNNKAERIVVIYLRDTPASMAERNQQIAGIGAALIEHWQR SHV-11_YMC2011/7/B774

241 IVALLGPNNKAERIVVIYLRDTPASMAERNQQIAGIGAALIEHWQR SHV-11_YMC2011/8/B10311

241 IVALLGPNNKAERIVVIYLRDTPASMAERNQQIAGIGAALIEHWQR SHV-11_YMC2010/8/B2027

**********************************************

**References for the table S1:**

Ahmed OI, Soha A EH, Tamer MA, and Iman ZA. 2013. Detection of bla SHV and bla CTX-M genes in ESBL producing Klebsiella pneumoniae isolated from Egyptian patients with suspected nosocomial infections. *Egyptian Journal of Medical Human Genetics* 14:277-283. doi:10.1016/j.ejmhg.2013.05.002

Bauernfeind A, Schneider I, Jungwirth R, Sahly H, and Ullmann U. 1999. A novel type of AmpC beta-lactamase, ACC-1, produced by a Klebsiella pneumoniae strain causing nosocomial pneumonia. *Antimicrob Agents Chemother* 43:1924-1931.

Bauernfeind A, Stemplinger I, Jungwirth R, Wilhelm R, and Chong Y. 1996. Comparative characterization of the cephamycinase blaCMY-1 gene and its relationship with other beta-lactamase genes. *Antimicrob Agents Chemother* 40:1926-1930.

C. Kamatchi HM, Uma Sekhar and Rama Vaidyanathan. 2009. Identification of Clonal Clusters of Klebsiella pneumoniae Isolates from Chennai by Extended Spectrum Beta Lactamase Genotyping and Antibiotic Resistance Phenotyping Analysis. *American Journal of Infectious Diseases* 5:74-82. 10.3844/ajidsp.2009.74.82

Chen TL, Lee YT, Kuo SC, Hsueh PR, Chang FY, Siu LK, Ko WC, and Fung CP. 2010. Emergence and Distribution of Plasmids Bearing the blaOXA-51-like gene with an upstream ISAba1 in carbapenem-resistant Acinetobacter baumannii isolates in Taiwan. *Antimicrob Agents Chemother* 54:4575-4581. 10.1128/AAC.00764-10

Dutour C, Bonnet R, Marchandin H, Boyer M, Chanal C, Sirot D, and Sirot J. 2002. CTX-M-1, CTX-M-3, and CTX-M-14 beta-lactamases from Enterobacteriaceae isolated in France. *Antimicrob Agents Chemother* 46:534-537.

Gonzalez Leiza M, Perez-Diaz JC, Ayala J, Casellas JM, Martinez-Beltran J, Bush K, and Baquero F. 1994. Gene sequence and biochemical characterization of FOX-1 from Klebsiella pneumoniae, a new AmpC-type plasmid-mediated beta-lactamase with two molecular variants. *Antimicrob Agents Chemother* 38:2150-2157.

Leiros HK, Borra PS, Brandsdal BO, Edvardsen KS, Spencer J, Walsh TR, and Samuelsen O. 2012. Crystal structure of the mobile metallo-beta-lactamase AIM-1 from Pseudomonas aeruginosa: insights into antibiotic binding and the role of Gln157. *Antimicrob Agents Chemother* 56:4341-4353. 10.1128/AAC.00448-12

Papanicolaou GA, Medeiros AA, and Jacoby GA. 1990. Novel plasmid-mediated beta-lactamase (MIR-1) conferring resistance to oxyimino- and alpha-methoxy beta-lactams in clinical isolates of Klebsiella pneumoniae. *Antimicrob Agents Chemother* 34:2200-2209.

Patzer JA, Walsh TR, Weeks J, Dzierzanowska D, and Toleman MA. 2009. Emergence and persistence of integron structures harbouring VIM genes in the Children's Memorial Health Institute, Warsaw, Poland, 1998-2006. *J Antimicrob Chemother* 63:269-273. 10.1093/jac/dkn512

Poirel L, Le Thomas I, Naas T, Karim A, and Nordmann P. 2000. Biochemical sequence analyses of GES-1, a novel class A extended-spectrum beta-lactamase, and the class 1 integron In52 from Klebsiella pneumoniae. *Antimicrob Agents Chemother* 44:622-632.

Queenan AM, Torres-Viera C, Gold HS, Carmeli Y, Eliopoulos GM, Moellering RC, Jr., Quinn JP, Hindler J, Medeiros AA, and Bush K. 2000. SME-type carbapenem-hydrolyzing class A beta-lactamases from geographically diverse Serratia marcescens strains. *Antimicrob Agents Chemother* 44:3035-3039.

Reisbig MD HN. 2002. The ACT-1 plasmid-encoded AmpC beta-lactamase is inducible: detection in a complex beta-lactamase background. *J Antimicrob Chemother* 49:557-560. 10.1093/jac/49.3.557

Sekiguchi J, Morita K, Kitao T, Watanabe N, Okazaki M, Miyoshi-Akiyama T, Kanamori M, and Kirikae T. 2008. KHM-1, a novel plasmid-mediated metallo-beta-lactamase from a Citrobacter freundii clinical isolate. *Antimicrob Agents Chemother* 52:4194-4197. 10.1128/AAC.01337-07

Sugumar M, Kumar KM, Manoharan A, Anbarasu A, and Ramaiah S. 2014. Detection of OXA-1 beta-lactamase gene of Klebsiella pneumoniae from blood stream infections (BSI) by conventional PCR and in-silico analysis to understand the mechanism of OXA mediated resistance. *PLoS One* 9:e91800. 10.1371/journal.pone.0091800

Toleman MA, Simm AM, Murphy TA, Gales AC, Biedenbach DJ, Jones RN, and Walsh TR. 2002. Molecular characterization of SPM-1, a novel metallo-beta-lactamase isolated in Latin America: report from the SENTRY antimicrobial surveillance programme. *J Antimicrob Chemother* 50:673-679.

Tzouvelekis LS, Tzelepi E, Mentis AF, and Tsakris A. 1993. Identification of a novel plasmid-mediated beta-lactamase with chromosomal cephalosporinase characteristics from Klebsiella pneumoniae. *J Antimicrob Chemother* 31:645-654.

Verdet C, Benzerara Y, Gautier V, Adam O, Ould-Hocine Z, and Arlet G. 2006. Emergence of DHA-1-producing Klebsiella spp. in the Parisian region: genetic organization of the ampC and ampR genes originating from Morganella morganii. *Antimicrob Agents Chemother* 50:607-617. 10.1128/AAC.50.2.607-617.2006

Yangsoon Lee B-SK, Jongsik Chun, Ji Hyun Yong, Yeong Seon Lee, Jung Sik Yoo, Dongeun Yong, Seong Geun Hong, Roshan DSouza, Kenneth S. Thomson, Kyungwon Lee, and Yunsop Chong. 2014. Clonality and Resistome Analysis of KPC-Producing Klebsiella pneumoniae Strain Isolated in Korea Using Whole Genome Sequencing. *BioMed Research International*:6. http://dx.doi.org/10.1155/2014/352862

Yong D, Toleman MA, Giske CG, Cho HS, Sundman K, Lee K, and Walsh TR. 2009. Characterization of a new metallo-beta-lactamase gene, bla(NDM-1), and a novel erythromycin esterase gene carried on a unique genetic structure in Klebsiella pneumoniae sequence type 14 from India. *Antimicrob Agents Chemother* 53:5046-5054. 10.1128/AAC.00774-09
